# Stability of exercise addiction symptoms and co-occurring mental disorders – a follow-up study

**DOI:** 10.3389/fpsyt.2025.1494309

**Published:** 2025-03-27

**Authors:** Maximilian Meyer, Aline Wagner, André Schmidt, Anna-Chiara Schaub, Undine E. Lang, Marc Walter, Flora Colledge

**Affiliations:** ^1^ University Psychiatric Clinics Basel, Clinic of Adult Psychiatry, University of Basel, Basel, Switzerland; ^2^ Faculty of Medicine, University of Basel, Basel, Switzerland; ^3^ Psychiatric and Psychotherapeutic Clinic, Psychiatric Services Aargau, Windisch, Switzerland; ^4^ Department of Health Sciences and Medicine, University of Lucerne, Lucerne, Switzerland

**Keywords:** exercise addiction, excessive exercising, clinical interview, behavioral addiction, compulsive exercising, exercise dependence, longitudinal data, diagnostic assessment

## Abstract

**Introduction:**

Individuals with exercise addiction (EA) report being unable to stop exercising despite its negative psychological, physical, or social impact. Due to a lack of evidence, EA has so far not been officially recognized as a behavioral addiction. Only one study exists, that investigated mental disorders in individuals with EA by utilizing the Structured Clinical Interview for DSM-5 Disorders (SCID-5). The present study followed up on this sample, providing the first longitudinal data on EA.

**Methods:**

All participants of the baseline study were invited to complete validated psychometric instruments assessing symptoms of depression, attention deficit hyperactivity disorder, trauma, and EA. Furthermore, an exhaustive diagnostic assessment with the SCID-5-CV, the SCID-5-PD (following a SAPAS screening), and a self-designed clinical interview to assess EA criteria were conducted.

**Results:**

The response rate was 59.4% (n=19) and the mean duration of follow-up was 634.5 (SD=155.8) days. Number of fulfilled EA criteria in the sample remained largely stable over time with no change in seven (36.8%), a reduction in nine (47.4%), and an increase in three (15.8%). Eighteen out of 19 participants fulfilled the criteria for at least one mental disorder. The most prevalent disorders were major depressive disorder (lifetime prevalence 73.7%; n=14) and obsessive-compulsive personality disorder (52.6%; n=10).

**Discussion:**

This data suggests that EA is a temporary stable and distinct disorder with affected individuals suffering from severe psychological distress. Further studies are necessary to investigate potential cause-effect relationships between co-occurring mental disorders and EA.

## 1 Introduction

Exercise addiction (EA) is a clinical phenomenon that was first reported on in the 1970s (1). It refers to a behavioral pattern comprised of rigid engagement in physical exercise, the inability to stop exercising despite its negative psychological, physical, or social impact, and experiencing psychological and physical distress when reducing or stopping physical exercise (2). To date, a variety of terms, among them “exercise dependence”, “compulsive exercise” and “excessive exercise”, have been introduced in the scientific literature to describe this behavioral pattern (3).

Unlike gambling disorder, EA has not yet been categorized as a behavioral addiction in the Diagnostic and Statistical Manual of Mental Disorders (DSM-5) (4). This is due to insufficient scientific evidence for the phenomenon; to date, no official diagnostic criteria exist (5). In the past ten years popular interest and case reports of EA have increased substantially (6). However, research conducted in the area suffers from a variety of methodological weaknesses (7). For the identification of EA, researchers have so far mostly relied on self-report instruments, with the Exercise Dependence Scale-21 (EDS-21) being one of the most widely used (8). This scale assesses EA, symptoms but has been demonstrated to overestimate the prevalence of EA if validated with clinical interviews (9). Furthermore, many of these measurement tools are unable to distinguish primary EA from instrumental or secondary EA (10). Whereas primary EA emerges independently from co-occurring disorders, instrumental EA serves the function of achieving a goal unrelated to exercise itself (11). This distinction is particularly relevant in case of co-occurring eating disorders, where exercise can be instrumental in controlling weight, but the physical activity itself is not directly rewarding for the affected individual (12).

Other co-occurring disorders in individuals with EA symptoms are depression, personality disorders, obsessive-compulsive disorders, anxiety disorders, ADHD, and substance use disorders (13–16). It is possible that exercise represents a maladaptive coping strategy for the affected individuals. However, comorbidities in these studies are usually derived from cross-sectional self-report surveys rather than structured diagnostic interviews conducted by clinicians. Hence, these studies do not allow inferences about the relationship between EA symptoms and comorbidities over time.

To date, the development of EA is not fully understood. Following the self-medication hypothesis, individuals suffering from these disorders might use exercise to alleviate symptoms of their “primary” condition (17). Likewise, EA could be understood as a symptom of the underlying disorder, rather than its own entity, thereby complicating diagnostic efforts. To provide a better understanding of the phenomenon and its aetiology, the interactional model of EA was proposed by Egorov and Szabo (2013) (18) and was expanded in 2021 (19). It provides a comprehensive framework, consisting of several intrinsic and extrinsic factors that may predispose or protect an individual from EA behavior in case of trigger events (described by the authors as “sudden or progressively intolerable life-stress”). Besides personal and situational factors, the interactional model considers a variety of underlying exercising motivations, explaining why some individuals might develop EA symptoms, whereas others express healthy exercising patterns. Since various paths may lead to EA, depending on life-circumstances and intrinsic factors, the interactional model explains the methodological hurdles previous EA researchers have encountered.

Currently, only one study has investigated mental disorders in individuals with EA by utilizing the Structured Clinical Interview for DSM-5 Disorders (SCID-5) (16). Meyer et al. recruited individuals who stated to exercise 10 h a week or more and continue their workout despite illness or injury. Flyers were distributed in gyms, universities, on public transportation, and via newspaper advertisements, inviting all persons between ages 18 to 70, who felt they did “too much exercise”, to contact the study group. These individuals were then asked to complete the Exercise Dependence Scale (EDS-21) (8), Beck’s Depression Inventory (BDI-II) (20), Homburger ADHS-Skalen für Erwachsene (HASE; Homburg ADHD Scale for Adults) (21), and the Childhood Trauma Questionnaire (CTQ) (22). Participants who met the EDS-21 cut-off for being “at risk for exercise dependence” (i.e., scoring 5 or 6 on three or more subscales) were subsequently invited to a second examination. In the second examination, all 32 participants completed a semi-structured interview about their exercising habits as well as the SCID-5-CV (23), which assesses all mental disorders aside from personality disorders. Furthermore, all participants completed the Standardized Assessment of Personality - Abbreviated Scale (SAPAS), a brief screening tool for personality disorders (24). In cases in which the SAPAS cut-off was met, the SCID-5-PD, which assesses personality disorders, was additionally employed. In total, 75% (n=24) of the sample fulfilled the diagnostic criteria for at least one mental disorder. The most common disorders in the sample were depressive disorders (56%), personality disorders (47%) and obsessive-compulsive disorders (31.3%) (16). However, the study followed a cross-sectional design and does not allow for conclusions about the stability of EA symptoms and co-occurring mental disorders over time.

By conducting follow-up diagnostic and EA interviews, this study is the first to report longitudinal data from the Meyer et al. sample of individuals with EA. This study aimed to investigate the stability of co-occurring mental disorders as well as EA symptoms and severity over time. No hypothesis was formulated prior to study conduction, as the goal of this exploratory study was to find out, whether EA symptoms increase, decrease, or remain stable over time. Elucidating the course of EA improves the understanding of the phenomenon. This would allow the improvement of diagnostic tools, as well as the development of targeted therapeutic interventions.

## 2 Materials and methods

### 2.1 Participants and procedure

A cross-sectional study was initiated in 2019, with the goal of identifying individuals who report possible EA symptoms (16). The present study followed up on participants of this study, who underwent the clinical interview process. All 32 individuals were contacted between twelve and eighteen months after completion of baseline data collection and invited to repeat completion of the baseline assessments (EDS-21, HASE, CTQ, and BDI-II) the interview process. The repeated use of these questionnaires was partly in order to ensure consistency over both measurement points, and partly as a manipulation check. Therefore, the follow-up EDS score was not used as a threshold below which certain participants might be excluded; all participants from baseline were interviewed if they agreed to follow-up, regardless of follow-up EDS score. Equally, the baseline inclusion criterion of exercising at least 10 hours per week did not apply for this study. This ensured that potential improvements in exercise addiction status were not overlooked due to the exclusion of participants, which would bias the narrative on the time-course of this disorder. Additionally, though assessments addressing past events like childhood trauma can be measured via a baseline screening and should not vary on repetition, we included all baseline screening measures again at follow-up to assess consistency of reporting, and flag possible discrepancies.

It is this follow-up study which we report on in this paper. All assessments were conducted through the online conference platform “Zoom”. Participants received CHF 150 for participating in the interviews.

### 2.2 Measures

All participants of the follow-up completed a brief semi-structured interview about their exercising habits and behavior. Participants were asked how their exercise habits developed and whether those habits changed over time. Moreover, participants were asked about their emotional state when exercise was impossible, and the impact of their exercising on their social life and physical health. An additional question inquired about changes in exercise habits since baseline study participation. The interview guide is provided in the **Supplementary Materials 1**. Exercise Addiction Symptoms – Interview Guide. The interview data were transcribed, coded, and compared to Colledge et al.’s 10 item symptom checklist (**Table 1**) to assess EA severity (2, 25). Grading spanned from subclinical (0-4 criteria), mild (5-6 criteria), moderate (7-8 criteria) to severe (9-10 criteria). Due to the brief nature of these interviews, no specific analysis framework for qualitative data was employed.

**Table 1:** Exercise addiction criteria by Colledge et al. used for severity grading (2).

1	Exercise volume has increased over time in order to avoid negative feelings of guilt or laziness
2	Negative affective response when exercise is reduced or sessions are missed/stopped
3	Attempts to reduce exercise volume are feared and/or unsuccessful
4	Preoccupation with exercise (e.g., having persistent thoughts of when and where next session will take place; planning workouts; thinking of ways to exercise during other activities)
5	Exercise is used as a way to cope with negative life experiences or stressors
6	Exercise is continued despite illness, injury or severe pain, at levels beyond rehabilitative training
7	Lying about or understating time and intensity of exercise
8	Jeopardising or losing significant relationships, jobs, educational opportunities, or career opportunities because of exercise
9	Continuing despite the rational understanding of negative physical and/or psychological burden of exercise habits
10	Feeling guilty when exercise is missed or reduced in volume

Diagnostic assessments with the SCID-5-CV were repeated for all follow-up participants (23). Participants who had been diagnosed with a personality disorder at baseline again completed the SCID-5-PD. All other participants completed the SAPAS and the SCID-5-PD was only conducted if the cut-off was reached (24). The SAPAS is a validated 8 item screening tool for personality disorders and the cut-off score of 3 correctly identifies the presence of a personality disorder with a sensitivity and specificity of.94 and.85 respectively (24).

Two participants reported a past episode of depression when interviewed at baseline, but did not mention this again at follow-up, a discrepancy likely due to erroneous reporting by participants. In these cases, we have taken the baseline assessment of major depressive disorder incidence and carried it forward.

### 2.3 Statistical analysis

All data was analyzed using SPSS version 26. No inferential statistics were calculated. Descriptive statistics calculated comprise means (M), standard deviation (SD) and percentages (%) and were employed for age, follow-up time, number of EA criteria, and number of diagnosed mental disorders. In addition, qualitative interview data was selected and summarized to characterize the study sample further.

### 2.4 Ethics

This study was approved by the responsible ethics committee (Ethikkommission Nordwest und Zentralschweiz) and conducted in accordance with the Declaration of Helsinki. All participants gave written informed consent before inclusion in the study.

## 3 Results

### 3.1 Sample description

All 32 participants of the baseline study were contacted, 19 (59.4%) of which responded and agreed to participate. The mean duration of follow-up was 634.5 (SD=155.8) days. Characteristics of the full sample are provided in detail in the baseline study (16).

The mean age of the sample (n=19) was 32.5 (SD=14.5) years, and 10 participants (52.6%) were female. **Table 2** provides an overview of fulfilled EA criteria and exercise volume in the current sample. **Table 3** shows the baseline and follow-up values of the screening questionnaires.

**Table 2:** Exercise addiction criteria and exercise volume in the sample.

		Baseline (n=19)		Follow-Up (n=19)	
		n	%	n	% (change)
EA severity	subclinical	3	15.8	3	15.8
	mild	5	26.3	7	36.8
	moderate	8	42.1	9	47.4
	severe	3	15.8	–	–
		M (SD)	MIN - MAX	M (SD)	MIN MAX
Number of fulfilled criteria		6.7 (2.5)	0 - 10	5.9 (1.7)	1 - 8
Exercise (hours per week)		16.9 (9.3)	5 - 45	13.6 (6.2)	5.3 – 25.0

subclinical: 0 – 4 criteria; mild: 5-6 criteria; moderate: 7-8 criteria; severe: 9-10 criteria.

**Table 3:** Development of screening questionnaire values.

	Baseline (n=19)	Follow-up (n=19)
	M (SD)	M (SD)
EDS-21	103.08 (13.86)	101.32 (14.59)
BDI-II	13.28 (8.10)	13.06 (7.85)
HASE	17.95 (12.45)	17.38 (13.49)
CTQ	57.63 (17.22)	58.41 (17.80)

EDS-21, Exercise Dependence Scale-21; BDI-II, Beck Depression Inventory; HASE, Homburger ADHS-Skalen für Erwachsene; CTQ, Childhood Trauma Questionnaire.

### 3.2 Natural history of EA symptoms

**Figure 1:**
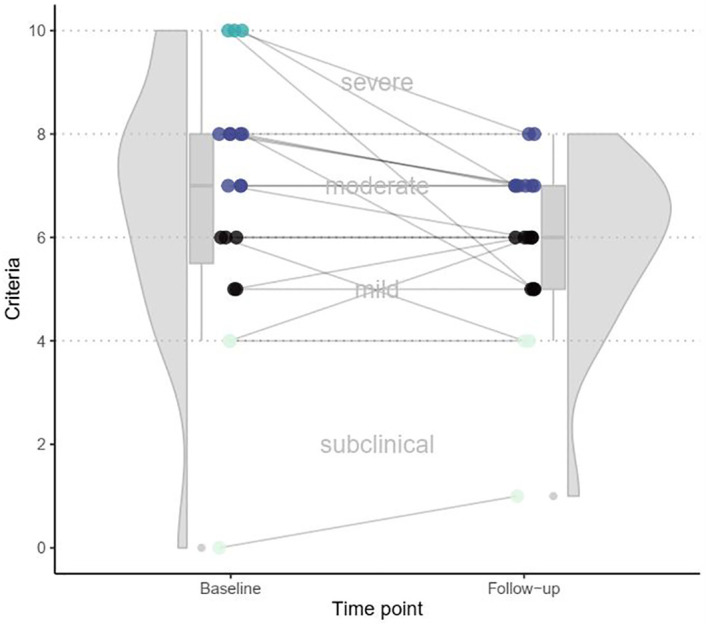
Development of fulfilled exercise addiction criteria over time.

Comparing the severity of EA on a case-by-case basis showed no change in seven (36.8%) participants. Nine (47.4%) participants fulfilled fewer EA criteria compared to baseline and three participants (15.8%) fulfilled more criteria compared to baseline. **Figure 1** presents each participants’ EA severity score as a connected baseline and follow-up datapoint.

### 3.3 Interview data

Most participants reported doing multiple sports. The most common were fitness/strength training (n=15), running (n=9), cycling (n=7), and hiking (n=5). Other disciplines included handball, skiing, swimming, volleyball, bouldering, rock climbing, triathlon, yoga, and cross-country skiing. In total, 6 subjects (31.6%) reported having professional ambitions or participating in competitions.

When asked about their feelings in case of missed workout sessions, participants reported feeling tense, irritable, angry, aggressive, out of balance, weak, and in a bad mood. Some participants stated to develop sleeping problems. Participants reported regulating their emotions with exercise and using it to cope with sensory overload, stress, unpleasant situations, and fights/arguments. One participant explicitly stated that exercise gives them the feeling of running away from their problems.

Some participants reported planning their social life around their exercising schedule. Skipping social events to exercise was a repeating theme, with friends and family showing a lack of understanding. One participant reported planning a sabbatical to be able to focus on sports fully, whereas another participant stated to have joined the military with the intention to make exercising/workouts their job.

When asked about physical problems related to their exercising, shoulder pain and inflammation, tendon ruptures, and hip- and knee joint problems were most commonly reported. Participants also commonly reported working around the affected body parts when exercising or not altering their workouts at all. One participant reported suffering from a double disc herniation, which forced them to switch to a more balanced workout regimen. Despite experiencing initial pain from a tendon rupture, another participant reported continuing with their exercising habits, noting that the pain had gradually decreased over time. A male participant reported suffering from a myocardial infarction recently, requiring four operations and multiple stents, forcing him to reduce his workout volume.

Participants were asked whether their exercising habits had changed since the baseline assessment. Most did not report a substantial increase or decrease in their workout volume. However, many participants reported that their gyms had closed because of COVID-19 lockdown regulations, leading to them increasing or picking up endurance sports like cycling or running. One participant reported becoming “more reasonable” and better able to cope in case of missing a workout session. Another participant reported substantially increasing their workout volume during the COVID-19 lockdown. Subsequently, they developed psychological and physical distress, which led to them seek psychiatric counselling and prescription of an antidepressant. This was accompanied by a span of nine months without any physical exercise, until they started exercising again.

### 3.4 Co-occurring mental disorders in the sample

Eighteen out of 19 participants fulfilled the criteria for at least one mental disorder. The total number of different mental disorders as identified by the SCID-5-CV and SCID-5-PD in the sample was 75. A comprehensive overview is provided in **Table 4**.

**Table 4:** Mental disorders in the sample as diagnosed through SCID-5-CV and SCID-5-PD interviews (n=19).

DSM-5 chapter	Disorder	Baseline n (%)	Follow-Up n (%)
Depressive Disorders	Major depressive disorder^a^	13 (68.4)	14 (73.7)
	Premenstrual dysphoric disorder	6 (31.6)	6 (31.6)
	Total (chapter)	19	20
Personality disorders	Any PD^b^	11 (57.9)	12 (63.2)
	Obsessive-compulsive PD	6 (31.6)	10 (52.6)
	Avoidant PD	4 (21.1)	2 (10.5)
	Narcissistic PD	2 (10.5)	1 (5.3)
	Schizoid PD	2 (10.5)	1 (5.3)
	Histrionic PD	1 (5.3)	–
	Paranoid PD	–	3 (15.8)
	Schizotypal PD	–	1 (5.3)
	Total (chapter)	26	30
Obsessive-compulsive and related disorders	Body dysmorphic disorder	2 (10.5)	–
	Hoarding disorder	2 (10.5)	1 (5.3)
	Excoriation disorder	3 (15.8)	3 (15.8)
	Obsessive-compulsive disorder	1 (5.3)	1 (5.3)
	Trichotillomania	–	1 (5.3)
	Total (chapter)	8	6
Anxiety disorders	Social anxiety disorder	4 (21.1)	3 (15.8)
	Generalized anxiety disorder	2 (10.5)	1 (5.3)
	Specific phobia	2 (10.5)	5 (26.3)
	Panic disorder	1 (5.3)	2 (10.5)
	Separation anxiety disorder	–	2 (10.5)
	Total (chapter)	9	13
Sleep-wake disorders	Insomnia disorder	5 (26.3)	5 (26.3)
	Hypersomnolence disorder	2 (10.5)	2 (10.5)
	Total (chapter)	7	7
Neurodevelopmental disorders	Attention-deficit/hyperactivity disorder	5 (26.3)	2 (10.5)
Substance-related and addictive disorders	Alcohol use disorder	2 (10.5)	–
	Stimulant use disorder	1 (5.3)	–
	Cannabis use disorder	1 (5.3)	1 (5.3)
	Other (Anabolic Steroids) substance related disorders	1 (5.3)	–
	Total (chapter)	5	1
Feeding and eating disorders	Anorexia nervosa	2 (10.5)	2 (10.5)
	Bulimia nervosa	1 (5.3)	2 (10.5)
	Avoidant/restrictive food intake disorder	1 (5.3)	1 (5.3)
		4	5
Trauma- and stressor-related disorders	Posttraumatic stress disorder	1 (5.3)	1 (5.3)
Somatic symptom and related disorders	Somatic symptom disorder	–	1 (5.3)
	Illness anxiety disorder	–	1 (5.3)
	Total (chapter)	–	2
Total number of disorders		73	75

PD: personality disorder; a. lifetime prevalence including recurrent and single episode; b. at baseline and follow-up 3 and 5 participants fulfilled criteria for multiple personality disorders respectively.

### 3.5 Individual changes in diagnosed psychiatric disorders

A total of six (31.6%) participants fulfilled the same number of psychiatric disorders at baseline and follow-up. The number of psychiatric disorders increased in seven (36.8%) participants and decreased in six (31.6%) (**Table 5**).

**Table 5:** Difference in number of psychiatric disorders as diagnosed through SCID-5-CV and SCID-5-PD interviews at baseline and follow-up.

Participant	Disorders at baseline	Disorders at follow-up	Difference
1	3	2	-1
2	4	5	+1
3	4	3	-1
4	8	11	+3
5	6	6	0
6	0	0	0
7	4	3	-1
8	4	5	+1
9	1	1	0
10	1	3	+2
11	4	4	0
12	6	1	-5
13	3	3	0
14	6	5	-1
15	11	6	-5
16	1	3	+2
17	2	2	0
18	5	9	+4
19	0	3	+3

### 3.6 Characteristics of participants lost to follow-up

When compared to the followed up sample, participants lost to follow-up on average fulfilled fewer EA criteria at baseline (M=5.8, SD=2.2 versus M=6.7, SD=2.5), had a lower average number of mental disorders (M=2.0, SD=3.1 versus M=3.84, SD=2.8) and spent more hours exercising per week (M=17.7, SD=11.9 versus M=16.9, SD=9.3).

## 4 Discussion

This is the first longitudinal clinical study of exercise addiction. The key finding of this study is that symptoms of EA remain largely stable over an average follow-up time of 1.7 years, adding weight to the increasing evidence that this is not a transient symptom cluster, but a distinct and persistent psychiatric disorder. Furthermore, the psychiatric comorbidities identified in affected individuals also remain broadly unchanged. Although participants in our sample suffered from severe psychological distress, the total number of 75 mental disorders present in a rather small sample may seem excessive. The amount is partly explained by us considering all DSM-5 disorders, including highly prevalent conditions such as a history of depression or insomnia (26).

Exercise addiction, like shopping, sex, or smartphone addiction, has received growing attention in recent years, as a society with increasing leisure time is exposed to these ostensibly harmless pastimes with greater frequency. As noted above, however, none of these phenomena is yet included in the non-substance-related disorder category of the DSM-5. As Potenza writes, this is due to the current lack of high-quality evidence for the characteristics of these potential disorders (27). In the case of EA in particular, it has been noted that current self-report questionnaires tend to lead to prevalence overestimates, and a general muddying of the waters surrounding this issue (28). Our study therefore addresses these methodological weaknesses on two fronts – by providing a detailed clinical picture of individuals reporting symptoms of EA, and a follow-up assessment to understand EA over a longer time frame.

Our results add yet another layer of evidence to the assertion that EA is indeed worthy of the name. Affected individuals report psychological distress caused by their behavior, yet feel unable to stop. The effects of exercise “withdrawal” ranged from feeling irritable and tense to the development of sleeping problems, which participants reported to avoid by organizing their social- and work-life around their workout regimen. Furthermore, exercise was continued despite the presence of physical injuries, which often likely directly resulted from their workout frequency and intensity. These apparent similarities to the behaviors observed in patients with substance use disorders underline the clinical significance of EA, while also providing reference points for clinicians aiming to treat the condition with targeted interventions in the future.

Almost two years later, just over half the sample reported no change or a worsening of symptoms, while 47% showed a reduction in symptoms. However, as shown in **Figure 1**, large increases or decreases in EA severity remained an exception in the sample. This is the first clinical evidence of the stability and persistence of EA symptoms.

It should be noted that the end of the baseline study and the entirety of the follow-up study took place during the first outbreak and repeated waves of the COVID-19 pandemic. While Switzerland had fairly relaxed regulations for the general public (no restrictions on time spent outside, while respecting social distancing), at numerous times gyms, pools and sporting goods stores were closed, and the practice of team sports was forbidden or limited. If EA were a subclinical, transient phase, one might expect a reduction in exercise-focused activity over this time period – the persistence of symptoms despite COVID-related challenges lends more weight to the claims regarding the severity of EA. In other countries, this time represented a period of significant distress that highlighted the compulsive nature of EA for affected individuals (29).

An important hurdle to overcome in the establishment of diagnostic criteria for EA is the occasionally-made suggestion that EA symptoms are not a distinct disorder, but are a subset of eating disorder symptoms, representing purging behavior. While the majority of the literature now accepts the distinction of primary and instrumental or secondary forms of EA, it would nevertheless change the nature of research on the topic if nearly all cases were observed in individuals with eating disorders (7). In the majority of our sample, this behavior is not explained by the primary goal of weight management or loss; with four diagnoses at baseline and five at follow-up, eating disorders were present in approximately a quarter of our sample. This is in line with other studies reporting instances of EA in the absence of any eating disorder (30). As a reason to exclude EA from the DSM-5, we feel that comorbidity with eating disorders can now be conclusively ignored. While future screening for other psychiatric comorbidities in individuals with EA is advisable, there is no grounds to expect a particularly high prevalence of eating disorders.

With regards to comorbid mental disorders, the most prevalent in our sample are major depressive disorder, and personality disorders, both of which appear in approximately 60% to 70% of the sample at both baseline and follow-up. As reported in an analysis of our original baseline sample, the depressive episodes generally pre-date the onset of EA, lending support to the claim that EA, like other addictive disorders, may be an attempt to self-medicate (31). Exercise is established as a rewarding behavior, and its effectiveness in reducing symptoms of depression matches that of pharmacotherapy (32). It is therefore intuitively plausible that the stability of depressive symptoms in our sample is matched by the stability in (potentially compensatory) EA. Notably, regarding compulsive personality traits, an increase from approximately 30% to 50% of the sample received a diagnosis of obsessive-compulsive personality disorder. This further supports the findings of a recent study, in which obsessive passion significantly predicted the occurrence of EA (33). Weinstein and Szabo also recently argued that EA lies within the obsessive-compulsive spectrum of behavioral addictions (7). In the literature, amongst the numerous synonyms for EA are “obsessive exercise” and “compulsive exercise”. These studies address the symptoms of panic and distress felt by individuals who cannot complete their planned exercise routine, as well as the perceived single-mindedness of pursuing opportunities to exercise (34). It is possible that exercising behavior originates as a way to reduce distress caused by obsessive-compulsive traits, with a negative spiral in some individuals leading to the same problematic symptoms affecting their exercise habits.

### 4.1 Strength and limitations

This study addresses several weaknesses of previous EA research. First, it provides longitudinal data, therefore allowing inferences about symptom stability over time. Second, it uses gold-standard diagnostic assessments. Third, it utilized in-depth semi-structured interviews rather than relying on measurement tools that have been shown to overestimate EA prevalence and severity. However, it must be noted that the findings are based on a small sample size, with numerous baseline participants lost to follow-up. This limits our results to being purely descriptive and future studies should attempt to employ inferential statistics, to test for the stability of exercise addiction symptoms and mental disorders. Furthermore, since SCID-5 interviews were conducted in an online video-conferencing format, their reliability may have been lowered.

### 4.2 Conclusion

This study is the first longitudinal clinical investigation of EA. The results suggest that EA is a temporally stable, distinct disorder. Furthermore, in this present sample, the numerous psychiatric comorbidities present at baseline also remained stable, suggesting that compromised mental health is a factor in EA. This data should provide a starting point for future clinical investigations of the potential cause-effect relationships between EA and comorbid psychiatric disorders.

## Data Availability

The raw data supporting the conclusions of this article will be made available by the authors, without undue reservation.
