# Supplementary material for: Stability of exercise addiction symptoms and co-occurring mental disorders – a follow-up study
Source: Front Psychiatry. 2025 Mar 27;16:1494309. doi: 10.3389/fpsyt.2025.1494309 (PMC11983396; doi:10.3389/fpsyt.2025.1494309)
Supplement: Supplementary file 1 [file Table1.docx]

Supplementary Material

# Exercise Addiction Symptoms – Interview Guide

INTRODUCTION

Describe your behaviour in your own words –is it perceived (by yourself and/or others) as a problem?

*(The following bullet-points and questions are asked, if they have not been addressed following the above prompt. Points which have been addressed are skipped (checked off by interviewer) and remaining points are specifically addressed.)*

- What types of exercise/sport/training do you do?
- How is the extent of it determined?
- Training plan (coach?) / rules / Rituals?
- Social contacts during/through training?
- Do you have specific goals you want to achieve with exercise?
- Is the programme flexible?
- What happens if something interferes? How do you feel?
- What happens if you are sick/injured?

EVOLUTION

- Since when have you had this problem/behaviour? (Start date?)
- Temporal development of the behaviour: When did you realise you had lost control over it? How did you notice?

CONSUMPTION PATTERNS

- Frequency of visits: How often per week/day do you engage in exercise/activity, and for how long each time? How much time do you spend daily on exercise/activity?
- Has there been any change over time (increase in dosage)? Have you noticed that you are training more often/longer/intensely?
- How much money do you spend in connection with your training? Are there debts or difficulties?
- How do you manage the compulsion/impulse to exercise?

CIRCUMSTANCES AND FUNCTION

- Do you sometimes hide your exercise habits?
- Are there specific situations where the impulse/desire to exercise becomes stronger or occurs unusually often?

CONSEQUENCES

- Effects on your environment? (e.g., effects on work, effects on relationships, etc.)
- Are there things bothering you about your exercising habits?

PREVIOUS TREATMENT ATTEMPTS

- Have there been attempts at abstinence? If yes, how many and how long?
- If yes, what circumstances led to the abstinence or supported it?
- If yes, what circumstances led to resuming the behaviour?
- Were there previous treatments? (Time period, place, what was done? Success?)

OUTLOOK

- What would appropriate exercise behaviour look like for you?
- Has your exercise behaviour changed since the last interview? If yes, how?
